# Association study of Glutathione S-Transferase polymorphisms and risk of endometriosis in an Iranian population

**Published:** 2016-04

**Authors:** Mina Hassani, Kioomars Saliminejad, Masood Heidarizadeh, Koorosh Kamali, Toktam Memariani, Hamid Reza Khorram Khorshid

**Affiliations:** 1 *Department of Biology, Faculty of Science, University of Kurdistan, Sanandaj, Iran.*; 2 *Reproductive Biotechnology Research Center, Avicenna Research Institute (ACECR), Tehran, Iran.*; 3 *Central Research Lab, North Khorasan University of Medical Sciences, Bojnurd, Iran.*; 4 *Genetic Research Center, University of Social Welfare and Rehabilitation Science, Tehran, Iran.*; 5 *Avicenna Research Institute, Shahid Beheshti University, Tehran, Iran.*

**Keywords:** *Endometriosis*, *Association study*, *GSTM1*, *GSTT1*, *GSTP1*, *Polymorphism*

## Abstract

**Background:**

Endometriosis influenced by both genetic and environmental factors. Associations of glutathione S-transferases (*GSTs*) genes polymorphisms in endometriosis have been investigated by various researchers; however, the results are not consistent.

**Objective:**

We examined the associations of *GSTM1* and *GSTT1* null genotypes and *GSTP1* 313 A/G polymorphisms with endometriosis in an Iranian population.

**Materials and Methods:**

In this case-control study, 151 women with diagnosis of endometriosis and 156 normal healthy women as control group were included. The genotyping was determined using multiplex PCR and PCR- RFLP methods.

**Results:**

The *GSTM1* null genotype was significantly higher (p=0.027) in the cases (7.3%) than the control group (1.3%). There was no significant difference between the frequency of *GSTT1* genotypes between the cases and controls. The *GSTP1* 313 AG genotype was significantly lower (p=0.048) in the case (33.1%) than the control group (44.4%).

**Conclusion:**

Our results showed that *GSTM1* and *GSTP1* polymorphisms may be associated with susceptibility of endometriosis in Iranian women.

## Introduction

Endometriosis is a multifactorial disease which is characterized by presence and growth of endometrial glands and stroma outside the uterine. It affects approximately 5-10% of reproductive age women in USA (1-3). Eidences of immunological, environmental, endocrine and genetic factors involved in endometriosis pathogenesis are available, however, its etiology and pathogenesis remain unknown (4, 5). Contribution of genetic factors to endometriosis pathogenesis was confirmed by higher risk of endometriosis among monozygotic twins compared to dizygotic twins as well as higher risk of recurrence in first-degree relatives (5, 6). 

genome-wide association studies showed that there is remarkable consistency in endometriosis (7). Several candidate genes in endometriosis including phase I and II detoxification genes, sex steroid pathways, cytokine signaling pathways, adhesion molecules and growth factor have been associated with endometriosis (8, 9). The glutathione S-transferases (GSTs) are the key phase II xenobiotic-detoxifying enzymes, which are upregulated in response to oxidative stress and overexpressed in many tumors (10, 11). The *GSTs* gene family encodes proteins that are critical for certain life processes, detoxication and toxification procedures. It is done by conjugation of reduced glutathione (GSH) with numerous substrates such as pharmaceuticals and environmental pollutants (11). 

To date, the most significant evidence linking specific polymorphisms to endometriosis comes from studies investigating phase II detoxification enzymes (9). *GSTM1* and *GSTT1*, two member of GST gene family, have null allele variants, which their homozygosity causes a complete lack of enzyme activity (12, 13). A consistent association of a GSTT1 polymorphisms and endometriosis, with a 29% increased risk of endometriosis in *GSTT1* null deletion carriers has been reported (9). The *GSTP1* 313 A/G polymorphism would result an amino acid substitution from isoleucine to valine at codon 105, which modifies the catalytic activity and heat stability of the enzyme (14). 

According to our knowledge, the results of *GSTP1* 313 A/G polymorphism and *GSTM1* null deletions with the endometriosis are not consistent. Accordingly, we investigated the association between *GSTM1*, *GSTT1* and *GSTP1* variations and susceptibility to endometriosis in Iranian women. 

## Materials and methods

**Subjects**

In this case-control study, 151 non-relative women with endometriosis were included, who referred to the Avicenna Infertility Clinic and Tehran Clinic Hospital, Tehran, Iran. The study protocol was approved by the Ethics Committee of the Avicenna Research Institute, and written informed consent was obtained from all participants. The diagnosis was made by visual inspection of the pelvis organs at laparoscopy, The sample size was estimated from previous study (15). 

Endometriosis women were classified to stage I to IV according to the revised American Society for Reproductive Medicine (ASRM) classification and they were found to have minimal (stage I), mild (stage II), moderate (stage III), and severe (stage IV) of endometriosis. The controls were 156 non-relative healthy women with no history of endometriosis and without any lesion as confirmed by laparoscopy. Because stage I and II of endometriosis are commonly found in asymptomatic women, therefore, in all controls absence of endometriosis was confirmed by laparoscopy (16). These people underwent laparoscopy for conditions other than endometriosis such as benign ovarian cyst. 

**Genotyping**

To investigate the association between *GSTM1*, *GSTT1* and *GSTP1* variations and susceptibility to endometriosis in Iranian women, the genotyping was performed by multiplex PCR and PCR-RFLP. Genomic DNA was extracted by salting out procedure from 5 ml of peripheral blood samples (17). Two multiplex PCR reactions were designed for the analysis of *GSTM1* and *GSTT1* null genotypes. The *ZFX* (495 bp) and *GAPDH* (113 bp) genes were used as internal controls in multiplex reactions containing *GSTM1* and *GSTT1*, respectively. Multiplex PCR technique could not distinguish between wild homozygous and heterozygous genotype of the *GSTM1* and *GSTT1* genes. 

**Figure 1:**
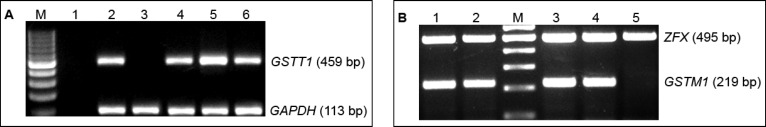
Results of *GSTT1* and *GSTM1* multiplex PCR on 1.5% agarose gels. A: Lane M: Marker 100 bp; Lane 1: DDW as negative control; Lane 3: *GSTT1* null deletion (Homozygote); Lane 2, 4, 5 and 6: At least on copy of GSTT1; GAPDH: as internal control. B: Lane M: Marker 100 bp; Lane 5: *GSTM1* null deletion (Homozygote); Lane 1-4: At least one copy of *GSTM1*; *ZFX*: as internal control

Accordingly, after electrophoresis the presence of *GSTM1* and *GSTT1* bands indicate that there is at least one copy of these genes. Each multiplex PCR reaction was performed in a final volume of 25 µl containing: 10 X PCR buffer, 1.5 mM MgCl2, 1U Taq DNA polymerase (CinnaGen, Iran), 0.5 mM dNTPs (Fermentas, Germany), 5 pmol of each primer, 50 ng template DNA, and sterile distilled water up to 25 µl. Amplification was performed with an initial denaturation at 94^o^C for 3 min, followed by 35 cycles of amplification which was performed at 94^o^C for 30 sec, 60^o^C for 30 sec, 72^o^C for 45 sec and a final extension at 72^o^C for 5 min. The PCR products were analyzed on 1.5% agarose gels and stained with ethidium bromide. The presence of a 459 bp or 219 bp bands indicated that there is at least one copy of the *GSTT1* and *GSTM1* genes, respectively, whereas the absence of these bands indicated the null genotype for these genes (Figure 1). The primer sequences and related product sizes are shown in table I.

**Fig 2:**
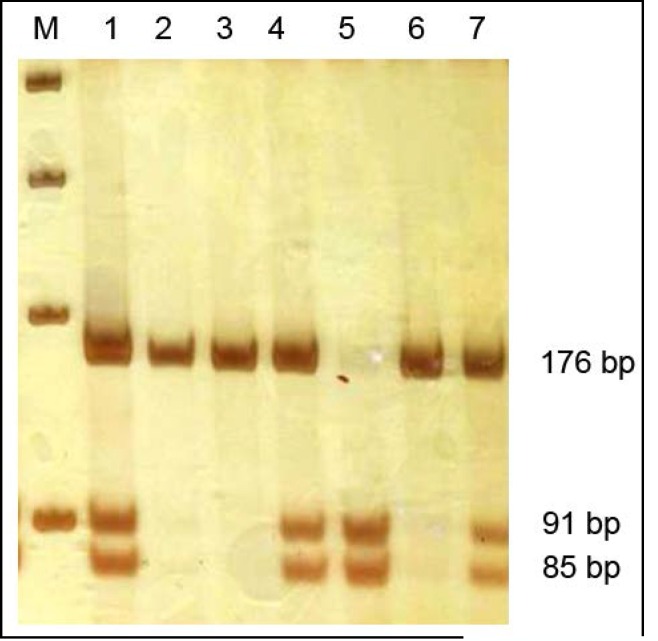
Results of RCR-RFLP for *GSTP1* 313 A/G polymorphism, separated on 10% polyacrylamide gel electrophoresis. Lane M: Marker 100 bp; Lane 2 and 6: PCR product as the control for digestion; Lane 3: Wild homozygote (AA); Lane 5: Mutant homozygote (GG); Lane 1, 4 and 7: Heterozygote (AG

The *GSTP1* 313 A/G polymorphism (rs1695) was analyzed by PCR-RFLP. The PCR amplification was carried out in a reaction mixture containing 10X PCR buffer, 2 mM MgCl2, 1U Taq DNA polymerase (CinnaGen, Iran), 0.5 mM of dNTPs, 5 pmol of each primer, 30 ng template DNA, and sterile distilled water up to 25 µl. Amplification was performed with an initial denaturation step at 94^o^C for 3 min, followed by 30 cycles at 94^o^C for 30 sec, annealing at 62^o^C for 30 sec and extension at 72^o^C for 30 sec, and a final extension at 72^o^C for 5 min. The PCR products of *GSTP1* were digested with the restriction enzymes BsmAI at 37^o^C overnight. The 313G allele produced two fragments with the length of 83 bp and 93 bp, while the 313A allele was not digested (176 bp). DNA fragments were subjected to 10% polyacrylamide gel electrophoresis and stained with silver nitrate (Figure 2). 

**Statistical analysis**

Statistical analysis was performed by IBM SPSS Version 20 (IBM Corporation, Chicago, IL, USA). Genotype and allele frequencies of each variation were compared between the case and control group by Fisher's exact test, Chi- square and logistic regression analysis. p<0.05 was considered statistically significant. All analyses were estimated by odds ratio and their 95% confidence intervals (CIs).

## Results

Descriptive analysis of 151 endometriosis women and 156 controls showed that the mean age of endometriosis and control groups were 31.4±6.0 and 29.3±5.3 years old, respectively. The mean BMI in the case and control groups were 25.0±4.7 and 25.6±5.6, respectively. The *GSTM1* null genotype was significantly higher (p=0.027, OR=5.76, 95% CI:1.22-27.11) in the cases (7.3%) than the control group (1.3%). 

This finding suggested that *GSTM1* null polymorphism may be associated with susceptibility to endometriosis. In the endometriosis group, homozygous women for the *GSTM1* null allele showed a six-fold increased risk of endometriosis than the controls (Table II). On the other hands, there was not a significant difference between the frequency of null and present genotype of *GSTT1* between the cases and controls (Table II). 

Genotype distribution in the control group for the *GSTP1* 313 A/G polymorphism was in Hardy-Weinberg equilibrium (p>0.05). Genotype and allele frequencies for *GSTP1* 313 A/G are summarized in table III. Our results showed that there was significant difference in the genotype distributions of *GSTP1* 313 A/G between the case and control groups. The *GSTP1* 313 A/G genotype was significantly lower (p=0.048; OR=0.61, 95% CI:0.37-0.99) in the case (33.1%) than the control group (44.4%). 

**Table I:** Primer sequences and their related sizes for each polymorphism

**Gene**	**Primer sequences (5′→3′)**	**Size (bp)**
*GSTP1* (27)	ACCCCAGGGCTCTATGGGAA	176 pb
	TGAGGGCACAAGAAGCCCCT	
*GSTT1* (27)	TTCCTTACTGGTCCTCACATCTC	459 pb
	TCACCGGATCATGGCCAGCA	
*GSTM1* (27)	GAACTCCCTGAAAAGCTAAAGC	219 pb
	GTTGGGCTCAAATATACGGTGG	
*ZFX*	ACCGCTGTACTGACTGTGATTACAC	495 pb
	GCACCTCTTTGGTATCCGAGAAAGT	
*GAPDH*	CCGGGTTCATAACTGTCTGC	113 pb
	TTCACACCCATGACGAACAT	

**Table II:** Genotype distribution and allele frequency of the *GSTM1* and *GSTT1* polymorphisms in endometriosis women and controls

**Gene**	**Genotype**	**Cases (n=151)**	**Controls (n=156)**	**p-value**	**OR (95% CI)**
*GSTM1*	Present*	140 (92.8%)	150 (98.7%)	Reference group	
	Null deletion	11 (7.3%)	2 (1.3%)	0.027**	5.76 (1.22-27.11)
*GSTT1*	Present*	117 (77.5%)	120 (76.9%)	Reference group	
	Null deletion	34 (22.5%)	36 (23.1%)	0.785	0.93 (0.53-1.62)

* There is at least one copy of wild allele

** Fisher’s exact test.

**Table III:** Genotype distribution and allele frequency of the GSTP1 313A/G polymorphism in endometriosis women and controls

**Genotype/Allele**	**Cases**	**Controls**	**p-value**	**OR (95% CI)**
AA	85 (56.3%)	72 (47.7%)	Reference genotype	
AG	50 (33.1%)	67 (44.4%)	0.048*	0.61 (0.37-0.99)
GG	16 (10.6%)	12 (7.9%)	0.863	0.93 (0.39-2.21)
A	220 (72.8%)	211 (69.9%)	Reference allele	
G	82 (27.2%)	91 (30.1)	0.418	0.864 (0.607- 1.23)

* Logistic regression (p< 0.05). n=151

**Table IV:** Summary of studies which evaluated the *GSTM1*, *GSTT1* and *GSTP1* variations in endometriosis. Positive and negative results abbreviated as P and N, respectively

**No.**	**Country**	**Study**	**Year**	**SNP**			**Case**	**Control**	**Race**
				***GSTT1***	***GSTM1***	***GSTP1***			
1	Russia	Baranov et al.	1996		P		42	67	Caucasian
2	France	Baranova et al.	1999		P		65	72	
3	UK	Hadfileld et al.	2001	N	N		148	148	
4	UK	Baxter et al.	2001		N		84	219	
5	Turkey	Ertunc et al.	2005			P	150	150	
6	Iran	Hosseinzadeh et al.	2011		P		120	200	
7	Iran	Seifati et al.	2012		N		101	142	
8	Italy	Vichi et al.	2012	N	N	N	181	162	
9	Brazil	Frare et al.	2013	P	P		50	46	
10	China	Peng et al.	2003		P		76	80	Asian
11	China	Lin et al.	2003	P	P		68	28	
12	Japan	Morizane et al.	2004	N	N		114	179	
13	Taiwanese	Hsieh et al.	2004		P		150	159	
14	South Korea	Hur et al.	2004	N	N	N	194	259	
15	South Korea	Jeon et al.	2010			N	260	194	
16	Japan	Matsuzaka et al.	2012	N	N	N	100	143	
This study			2015	N	P	P	151	156	Caucasian

## Discussion

The aim of this study was to evaluate whether the *GSTM1*, *GSTT1* null genotypes and *GSTP1* 313 A/G polymorphism are associated with susceptibility to endometriosis. In Caucasians, the frequencies of homozygous deletions of *GSTM1* and *GSTT1* are approximately 50% and 10-20%, respectively. Most studies investigating the effect of *GSTM1* and *GSTT1* null polymorphisms do not distinguish between individuals with one or two copies of the genes; therefore, the effects of functional gene dosage could not be explored (17). The *GSTP1* 313 A/G polymorphism (Ile105Val at codon 105), resulting in an enzyme with altered substrate affinity. Approximately 10% of Caucasians are homozygous for this mutation and 40% are heterozygous (18).

Our results showed that the *GSTM1* null genotype might be associated with the risk of endometriosis in Iranian women. The endometriosis women with *GSTM1* homozygous null genotype had a six- fold increased risk of developing endometriosis (p=0.027; OR=5.76). Also, *GSTP1* 313 A/G polymorphism was associated with the endometriosis, however, 313 A/G genotype had a protective effect (p=0.048, OR=0.61), which decreases the risk of the disease. In contrast, no significantly differences between the *GSTT1* null deletion and endometriosis was observed. To compare our findings, we search the PubMed database for studies that examined the association between *GSTM1*, *GSTT1*, and *GSTP1* 313 A/G polymorphisms with endometriosis up to July 2014 (Table IV). 

Altogether, we found 15 publications, in which nine and eight studies were performed in Caucasian and Asian populations, respectively. Briefly, positive and negative results were found in seven and six of these studies, which evaluated the *GSTM1* null genotype and endometriosis, respectively (18-23, 25-30). Only one out of five studies, in a Turkish population, reported that there is a positive association between *GSTP1* 313 A/G and endometriosis (14, 27, 28, 30, 31). Two out of seven publications found an association between *GSTT1* null genotype and the disease (21, 23, 26-30). Positive association of *GSTM1* null genotypes and endometriosis in our study is consistent with the results of Hosseinzadeh *et al* which performed an association study in an Iranian population (21). In contrast, in another study in Iranian population by Seifati *et al*, no association was found between this null polymorphism and endometriosis (32).

As table IV clearly shows, the results of association studies in different populations are inconsistent, which could be attributed to small sample sizes, poorly matched control group and heterogeneity within populations. Because minimal and mild stages of endometriosis may be found in asymptomatic women, therefore, in control group absence of endometriosis should be confirmed by laparoscopy (16). However, Morizane *et al* used umbilical cord blood from female newborn infants as population controls for an association study of *GSTM1* and *GSTT1* variations in women with endometriosis in a Japanese population (29). If the case and control groups are not well matched for ethnicity or geographic origin then false positive association may be occurred because of the confounding effects of population stratification (33). 

Two meta-analysis evaluated the glutathione S-transferase variations in endometriosis women (13, 34). Guo *et al* performed a meta-analysis involving 14 studies investigating *GSTM1* and nine *GSTT1* studies. The results of their analysis demonstrated an association of *GSTT1* polymorphism and endometriosis, with a 29% increased risk of endometriosis in individuals homozygous for *GSTT1* null genotype. They found no evidence that women with *GSTM1* null genotype have increased risk of developing endometriosis (13). However, there was evidence of publication bias in this meta- analysis, indicating that the size of the increased risk associated with the *GSTT1* deletion variant may actually be smaller or non- existent (9). In a recent meta- analysis by Chen *et al* they concluded that the *GSTP1* 313 A/G may not be associated with endometriosis risk (34). 

In conclusion, the *GSTM1* and *GSTT1* null variations may be associated with the increased risk of endometriosis in Iranian population. Additional studies on different populations are necessary to further confirm the role of glutathione S-transferase variations in the pathogenesis of endometriosis.
